# Measuring equity of access to eye health outreach camps in rural Malawi

**DOI:** 10.1371/journal.pone.0268116

**Published:** 2022-05-20

**Authors:** Guillaume Trotignon, Thomas Engels, Shaneez Saeed Ali, Ziporah Mugwang’a, Iain Jones, Stevens Bechange, Effie Kaminyoghe, Tesfaye Haileselassie Adera, Elena Schmidt

**Affiliations:** 1 Sightsavers, Haywards Heath, West Sussex, United Kingdom; 2 Sightsavers, Nairobi, Kenya; 3 Sightsavers, Lilongwe, Malawi; 4 Sightsavers, Addis Ababa, Ethiopia; University of Washington, UNITED STATES

## Abstract

**Introduction:**

Equity in the access and use of health services is critical if countries are to make progress towards universal health coverage and address the systematic exclusion of the most vulnerable groups. The purpose of this study was to assess if the Co-ordinated Approach To Community Health programme implemented by Sightsavers was successful in reaching the poorest population, women, and people living with disabilities in Kasungu district, Malawi.

**Methods:**

Between April and September 2017, data on socio-economic status, household characteristics and functional disability were collected from patients attending at eye camps in Kasungu district, Malawi. Using asset-based tools to measure household wealth (EquityTool© and Simple Poverty Scorecard©) and the Washington Group Short Set of Questions, individuals were categorised by wealth quintiles, poverty status, and functional disability status and then compared to relevant representative national household surveys. In addition, a follow-up household survey was conducted to check the validity of self-reported household characteristics at eye camps.

**Results:**

A total of 1,358 individuals participated in the study. The study shows that self-reported data on household characteristics and assets are reliable and can be collected in clinical settings (instead of relying on direct observations of assets). Individuals attending outreach camps were poorer in terms of relative wealth and absolute poverty rates compared to the rest of the population in Kasungu. It was estimated that 9% of the participants belonged to the poorest quintile compared to 4% for the population in Kasungu (DHS 2015–2016). The ultra-poverty rate was also lower among respondents (13%) compared to 15% for Kasungu district (IHS 2017). The functional disability rate was 27.5% for study participants, and statistically higher than the general population (5.6%, SENTIF 2017). Even though women are more at risks than men, 54% of the participants were men.

**Conclusions:**

Our study shows that existing tools can be reliably used, and combined, if based on recent population data, to assess equity of access to health services for vulnerable groups of the population. The findings suggest that the programme was successful in reaching the poorest people of the Kasungu district population as well as those with disabilities through outreach camps but that more men than women were reach through the programme. Subsequently, our study showed that self-reported household characteristics are a reliable method to measure asset-based wealth of camps’ attendee. However, it is essential to use sub-national data (district or regional level) from recent surveys for the purpose of benchmarking in order to produce accurate results.

## Introduction

Universal health coverage is central to achieving the 2030 Sustainable Development Goals (SDGs) and ensuring no one is left behind [1]. In recent years, there has been good progress in the coverage of some essential interventions, whilst the coverage for others remains low both geographically and in population sub-groups [2]. In this context, there has been a growing interest in measuring equity of access to health care and understanding who is being left behind [3, 4]. Equity in healthcare should improve disparities in access amongst communities of different socio-economic contexts and can be achieved through addressing the systemic factors that result in marginalization [5, 6].

Many development programmes make an assumption that targeting rural and remote areas is by virtue equitable, as the poverty levels and marginalisation are particularly high in such locations [7]. However, even in remote and rural parts of a country, interventions may only reach and improve health outcomes for individuals who are comparatively advantaged [8]. Without assessing the socio-demographic and socio-economic profile of programme participants, resources may not be allocated to those in greatest need leading to an unintentional widening of the health gap.

Eye health is one of the areas where coverage with essential interventions continues to be sub-optimal, particularly in Sub-Saharan Africa (SSA), where over 21 million people live with visual impairments, including 4.3 million, who are blind [9].

Data on eye health service coverage in different population sub-groups are rare. Rapid Assessments of Avoidable Blindness (RAABs), which are the main source of population-based data on visual impairment, only recently started collecting data on variables other than sex and age [10]. Data from the studies available show that women, people from poorer households and those with additional (non-visual) disabilities are disadvantaged in accessing eye care services. There are also context-specific differences which are difficult to review systematically as only a few RAABs have these more comprehensive data.

In this context, routinely collected data from eye health programmes could be a valuable source of information about equity in eye health [11]. However, at present eye health services rarely collect data on patient characteristics beyond sex and age and even these basic data are not always used to assess equity of service delivery.

A recent paper by Evans et al. reviewed equity in the studies included in the Cochrane Eye and Vision systematic reviews [12]. The authors identified 62 unique sources, of which only two reported data by socio-economic status, two by place of residence, three by education and one by occupation. Sex-specific data was reported in the majority (73%) of papers, but the sub-group analysis of sex data was included in only two studies. The authors highlighted the urgent need to prioritise equity data in eye health research.

The study presented here was integrated in an eye health programme called the Co-ordinated Approach To Community Health (CATCH). The CATCH programme delivered eye care services, such as cataract, refractive error, and conjunctivitis through outreach camps, where patients with minor morbidities were treated on-site; and those with more complex conditions were taken to a nearby hospital. It took place in the Kasungu district in the Central Region of Malawi.

The main objective of the study was to assess equity of access to CATCH camps’ eye care services, aiming to answer the question: is the project reaching out to the poorest and equally to person with disabilities and women? To answer this question, three subsequent objectives were defined: 1) Validation of self-reported household characteristics; and 2) Comparison of CATCH beneficiaries’ socio-economic status with the rest of the district population using questionnaire’s results. It was anticipated that the data would be relevant and useful to the programme to guide its community mobilisation and awareness raising campaigns, thereby ensuring those in the greatest need were reached and no one in the target community was left behind.

## Methods

### Study design and population

**Fig 1:**
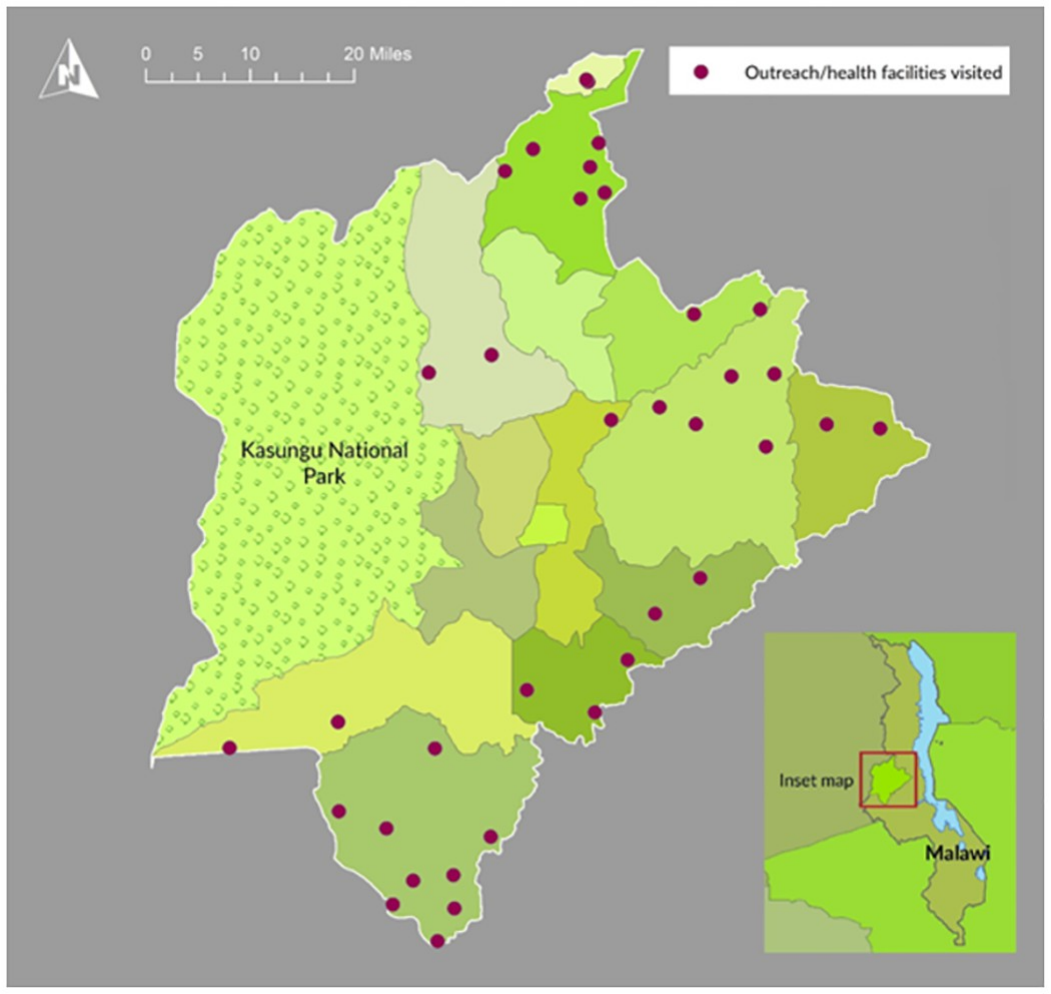
Geomap of health facilities visited. Republished from Chatharoo et al. 2018 under a CC BY license, with permission from Andy Tate, Senior Data Management and Reporting Advisor, Sightsavers, original copyright 2018 [13]. Base maps were obtained from the GADM database of Global Administrative Areas (http://www.gadm.org/).

The study design was a cross-sectional survey of patients attending eye health outreach camps in the district of Kasungu, central Malawi (Fig 1).

Study participants were recruited from those who attended the outreach camps, providing they were over the age of 18 years, or accompanied by an adult if below this age and gave consent.

The study was conducted between April and September 2017. During this period eye camps were organised monthly across the district and lasted between two and four days each (Fig 1). Comprehensive eye health activities were delivered alongside outreach camps targeting trachoma trachomatous (TT) and organised as part of the trachoma elimination programme funded by the Queen Elizabeth Diamond Jubilee Trust (QEDJT) [14].

### Study tools

#### Measuring participants’ socio-economic status

Income is commonly used as a measure of socio-economic status. However, it is extremely difficult to measure income in low- and middle-income contexts with large informal sectors and where income does not include in-kind payments and/or fluctuates according to seasonality and migration [15]. Household ownership of assets can be used as an alternative to estimate wealth and poverty levels in such contexts [16–18]. However, the measurement of patients’ socio-economic status via asset-based wealth indexes are usually conducted through household surveys Therefore, the validity of self-reported asset-based wealth assessment had to be tested (see Sampling and Data analysis sub-sections).

In this study we used two validated asset-based tools, the EquityTool and the Simple Poverty Scorecard [19, 20].

The Simple Poverty Scorecard, developed by Mark Schreiner of Microfinance Risk Management L.L.C, is a country specific tool, which estimates the likelihood of a household’s consumption to be below a certain poverty line, based on their characteristics and asset possession. The Malawi tool available at the time of this study consisted of 10 key indicators, based on the Malawi’s 2010/2011 Integrated Household Survey (IHS) [20, 21].

Each response to an indicator has a given number of points, and the total “poverty score” for a household ranges between 0 and 100 points. The calculated poverty score for each household is then compared to a matrix to estimate the poverty rate, or the proportion of a group to be below four government defined poverty lines: i) food or ultra-poverty line; ii) national poverty line (includes food and non-food components); iii) $1.90 per day line; and iv) $3.10 per day line (20, 21).

The EquityTool, developed by Metrics for Management, measures relative wealth based on household characteristics and possession of durable assets. The Malawi EquityTool 2015 is composed of 17 indicators, and the corresponding scores are computed through a principal component analysis based on the Malaria Indicator Survey from 2012 [19].

Each DHS participant is given a wealth index according to the 17 indicators generated and are then ordered and split into five equal quintiles based on their score, Quintile one represents the poorest segment of the population, and quintile five the wealthiest. Using national quintiles to determine cut-off points (based on the score of the 17 indicators), our study participants are allocated to their corresponding quintile, which allows a comparison of their socio-economic status with the rest of the national population. Indeed, if study participants’ level of wealth is the same as the national population, each quintile would have 20 percent of respondents. If the level of wealth is different, the five quintiles would be unequally split. The study used the latest version of the tool available at the time of the study which was validated for Malawi based on the Malawi MIS 2012 [19, 22].

Both national surveys (IHS and MIS), used for deriving tools’ indicators, allow for disaggregating data at the regional and district level. Therefore, in our analysis for both tools we compared wealth of our programme participants with the national population and the population of Kasungu district. In addition, by the time of the data analysis two new national surveys (DHS from 2015/16 and IHS 2016/17) had been released [23, 24]. We therefore used the same indicators for both tools with these more recent datasets.

#### Measuring disability

The Washington Group Short Set of Questions on Disability (WGSS) was used to measure self-reported disability. The tool was developed by the United Nations Statistical Commission for use in national censuses and surveys. The tool assesses functional difficulties when conducting basic activities in six domains: seeing, hearing, walking/climbing; remembering/concentrating; self-care and communicating. The answers are given on a four-point scale from ‘no difficulty’ to ‘cannot do at all’. Disability status is defined when participants report having a lot of difficulty or cannot do at all in at least one domain [25]. As we expected, a significant number of participants coming to the outreach camps had difficulty in seeing. We also used a measure of “non-visual disability”, i.e., a functional difficulty (a lot of difficulty or cannot do at all) in any domain except seeing.

### Sampling

The study population was all individuals who presented at outreach camps who met the inclusion criteria stated above. Three camps have been randomly selected, where all patients coming for eye examination were systematically interviewed, upon consent. Five data collectors worked for 24 days until sample sized requirement was reached.

The sample size was calculated based on the formula for proportions’ comparison [26]. The following assumptions from an earlier pilot and from Demographic and Health Surveys (DHS) 2015/2016 were used: i) an estimated prevalence of non-visual disability of 10%; ii) 8% of those with a disability would belong to the wealthiest quintile; and iii) a power of 80% to detect a 10% difference in the prevalence of disability between attendees belonging to the poorest group (quintile 1) and the wealthiest group (quintile 5) at alpha 0.05, with a ratio of 2. The minimum sample size required was 1,275 participants, and 1,358 observations were collected in total [23]. In this study household and dwelling data were self-reported. Therefore, a number of household visits were organized for randomly selected patients participating in the study in order to verify the validity of these data and tackle the first sub-objective. The sample size needed for the household visits was calculated to be 102, using a 7.5% margin of error (alpha 0.05), and 156 households had been selected for data verification, again upon consent.

### Data collection

Upon arrival at the screening site, all attendees were provided information about the study by the data collection team. Information was provided in local languages and people had an opportunity to ask questions.

**Fig 2:**
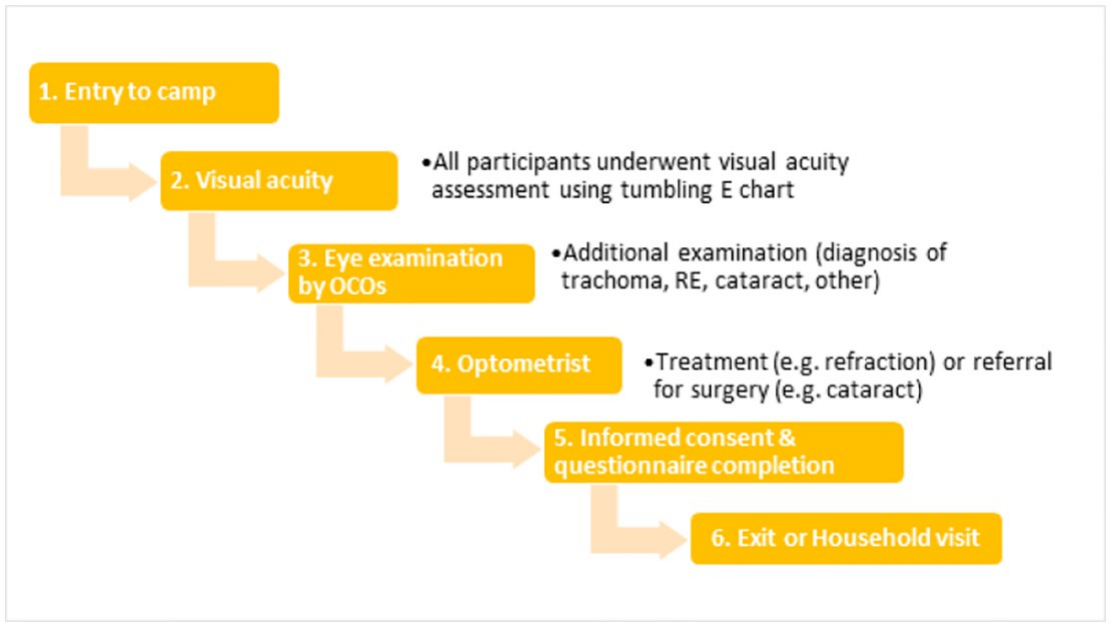
Flow diagram of outreach camp process.

All attendees first undertook visual acuity test using a tumbling E chart. Those who failed the test or had other visible eye problems (such as red eye) were examined by the Ophthalmic Clinical Officer, who made a diagnosis and provided a treatment or a referral. Patients selected by the interval random sampling (see above) for a further interview were again informed about the study, its purpose and how the data will be used and were asked to provide their consent. If provided, they were asked questions from the EquityTool, Simple Poverty Scorecard and WGSS questionnaires. A subset of participants was then randomly selected for home visits to verify their household asset scores (Fig 2).

The data was collected electronically using the mobile phone survey software KOBO ToolBox [27]. Five data collectors with experience of collecting mobile data were recruited and trained ahead of the camp. The questionnaires were administered in local languages, Chichewa and Tumbuka (S1 Appendix).

Household visits to verify self-reported wealth were attempted on the same day as the camp visit when possible; if not, they were carried out the following day. Community volunteers assisted the data collectors to trace the respondents’ homes. All data are available in S1 Dataset for camp-based data collection and in S2 Dataset for Kappa analysis (S1 and S2 Datasets).

### Data analysis

Personal identifiable information collected was separated from the rest of the data before the analysis. Data cleaning and analysis was undertaken using STATA 14 [28].

Kappa statistics were used to measure the inter-rater reliability of self-responses at the camp compared to the household survey on dwelling characteristics and ownership of assets, following the methodology described in the literature [29]. The guidelines from Landis & Koch (1977) were used to interpret the level of agreement as follows: 0.0–0.20: slight; 0.21–0.40: fair; 0.41–0.60: moderate; 0.61–0.90: substantial; 0.81–1.00: almost perfect [30].

To compare the level of wealth of camp participants to the rest of the Kasungu district, both MIS 2012 and DHS 2015/2016 data sets were retrieved from the Demographic and Health Surveys programme website and were compared against our study sample. For the analysis using DHS 2015/16 data, the EquityTool 2010 wealth index was used, similar to methods used by Pitchforth et al. and Wilunda et al. [19, 31–33]. The choice of applying the EquityTool proxy variables 2010 on the DHS 2015/16 data was justified by the fact that there was an important time gap between MIS 2012 and DHS 2015/16, and that the updated Malawi EquityTool 2017 contained different questions that our survey did not cover (S1 Fig) [34]. The same procedure was applied to the Simple Poverty Scorecard score (based on 10 indicators as seen above) using the IHS 2011 and IHS 2017 at national and district levels as references [21, 24].

STATA complex design-based F-test of independence was used to test our hypothesis that the proportion of study participants belonging to the lowest relative wealth quintile was different from the Kasungu residents, the MIS, and from the DHS full sample [35].

STATA 14 was also used to assess participants’ disability status using the recommended cut off (a lot of difficulty or cannot do at all) in at least one domain. Non-visual disability was determined by using the same cut offs but excluding the seeing domain. Disability data from the SINTEF national survey, conducted in 2017 and using the same WGSS tool, were compared to CATCH camps participants [36]. A chi-square test of independence was performed for 10-year age groups (except for the 0 to 20 years old) in order to have homogenic, large enough sub-groups. This also corrected for the age distribution difference given that disability is positively associated with age [37–39].

### Ethical considerations

Ethical approval was obtained from the Malawi National Health Sciences Research Committee (NHSRC) [protocol #16/11/1685]. Informed consent was obtained from all study participants. In the case of minors, their parent, or legal guardian, provided the consent. Verbal consent was first requested at camp level, prior to household visits; and at the household level, again a verbal informed consent was sought from both the head of the household and the study participant (S2 Appendix). All information collected was anonymised and kept confidential. All study participants with eye problems were either treated at the camp or referred to nearby surgical services.

## Results

### Participants’ characteristics

Over a five-month period, 1,358 participants were recruited at the eye camps and participated in the study. Table 1 summarises demographic characteristics of participants.

**Table 1:** Sociodemographic characteristics of study participants (n = 1,358).

Socio-demographic characteristics		N (%)
Sex		
	Men	733 (53.98)
	Women	625 (46.02)
Age group		
	<18	136 (10.01)
	20–29	130 (9.57)
	30–39	190 (13.99)
	40–49	257 (18.92)
	50–59	231 (17.01)
	+60	414 (30.49)
Marital status		
	Married/partnership	1,025 (75.48)
	Divorced/separated	66 (4.86)
	Never married	75 (5.52)
	Widowed	192 (14.14)
Education level		
	No education	211 (15.54)
	Primary	823 (60.60)
	Secondary	290 (21.35)
	Higher than secondary	34 (2.50)
Occupation		
	Agriculture	1,010 (74.37)
	Service worker	16 (1.18)
	Sales worker	53 (3.90)
	Production worker	38 (2.80)
	Professional	115 (8.47)
	Unemployed/student/other	126 (9.28)
Frequency of work		
	All year	515 (41.63)
	Seasonal	687 (55.54)
	Occasional	35 (2.83)

There were more men than women in the sample (54% vs 46%). Around half of the participants (47.5%) were aged 50 years and above with both the mean and median age of 49 years. Most participants were married (75%). Sixty one percent had attained primary school education only, while about one fifth (21%) had received secondary education; and 16% had no education. Only 34 participants (2.5%) had education beyond secondary.

The majority (74%) reported agriculture as their main occupation followed by professional skilled jobs i.e. technical, administrative, managerial roles (8%). Around 56% said that their occupation was seasonal, while 42% said they worked all year round.

### Participants’ socio-economic status

#### Validity of self-reported data

Table 2 shows that there was a high degree of agreement (80%) between household wealth based on self-reported characteristics and assets and those observed during the household visits with a kappa statistic of 0.74. The finding confirms the accuracy of the self-reported estimates collected during the camps (see S1 Table).

**Table 2:** Household and camp inter-rater reliability.

	Agreement (%)	Expected agreement (%)	Kappa	Standard error
Household versus camp responses	80.1%	24.7%	0.7361	0.0438

#### Simple poverty score card results

Our data shows that 13.2% of the camp attendees were below the ultra-poverty line (also called food line with a threshold of 2,400 calories per day); 35% were below the national poverty line; 65% were below the $1.90 threshold, and 84% were below the $3.10 a day threshold (Purchasing Power Parity 2011). When compared to the national and Kasungu populations in 2011, our participants were on average wealthier. For example, the respective poverty rates in Kasungu district were 19.8%, 44.6%, 73.1% and 88.9% in 2011. As indicated in Table 3, absolute poverty rates in Kasungu and at the national level have decreased over the period 2011 and 2017, and poverty rates among camp attendees are similar to poverty rates calculated for Kasungu district in 2017 (IHS 2017).

**Table 3:** Poverty rates using government. Defined thresholds (2011 poverty lines).

Government poverty lines	Percentage of individuals leaving in a household with a consumption below a poverty line				
	Camps participant (2017) (n = 1,358) % (95% confidence interval)	IHS 2011–Kasungu (n = 384)* % (95% confidence interval)	IHS 2011–National (n = 12,271)* % (95% confidence interval)	IHS 2017–Kasungu (n = 384)* % (95% confidence interval)	IHS 2017–National (n = 12,447)* % (95% confidence interval)
Ultra-poverty line	13.2 [12.4–13.9]	19.8 [17.8–21.8]	19.9 [19.5–20.3]	14.2 [12.7–15.6]	11.5 [11.2–11.8]
National poverty line	35.0 [33.6–36.3]	44.6 [41.7–47.5]	44.3 [43.7–44.9]	37.2 [34.6–39.6]	31.6 [31.1–32.1]
$1.90 dollar per day, PPP 2011	65.2 [63.8–66.5]	73.1 [70.6–75.8]	72.2 [71.6–72.7]	67.3 [65.0–70.0]	60.9 [60.4–61.5]
$3.10 dollar per day, PPP 2011	84.0 [83.0–85.1]	88.9 [87.1–90.7]	87.8 [87.4–88.2]	85.6 [84.0–87.7]	80.9 [80.5–81.4]

*Weighted poverty rates

**Fig 3:**
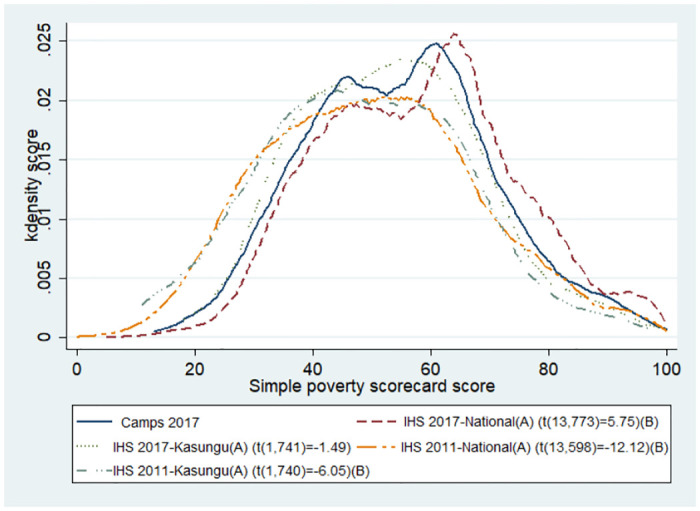
Simple poverty scorecard results, comparison by sample. (A) Weighted using IHS household sampling weights. (B) STATA survey design adjusted samples t-tests of independence with camp participants, significant at alpha = 0.001 (two tails).

Fig 3 graphically represents the poverty scores of camp attendees compared to the national and Kasungu populations in 2011 and 2017. The statistical tests using Stata survey design adjusted samples t-test also confirmed that the camp attendees were wealthier than the Kasungu and national population in 2011 (respectively t(1,740) = -6.05, p = 0.000 and t(13,598) = -12.12, p = 0.000). On the other hand, the national sample of the IHS 2017 had a statistically higher mean score (t(13,773) = 5.75, p = 0.000). Finally, no statistical difference were observed between camps’ mean score and Kasungu population of 2017 (t(1,741) = -1.49, p = 0.137).

#### EquityTool results

Looking at the wealth quintiles in the sample using the MIS 2012 cut off points (Table 4), the results suggest that our participants were relatively wealthier than the rest of the country, as over half of the participants belonged to the two wealthiest quintiles and only 25% to the two poorest quintiles. There is an even greater gap with the Kasungu population in 2012, which was poorer than the national population and where 55% of residents belonged to the two poorest quintiles and only 28% to the two wealthiest quintiles. However, when we compare the 2015–16 DHS data with the MIS 2012 data, we can see that the 2015–16 populations are wealthier, as over 53% in Kasungu and 62% nationally belong to the two wealthiest quintiles using the 2012 thresholds. In fact, camp attendees appear to be poorer than the national and Kasungu populations in 2015–16, as 25% of our sample belonged to the two poorest quintiles compared to only 12% in the national and Kasungu 2015/16 populations. (Table 4). Our statistical analysis also confirmed these observations, as a significant difference in terms of quintiles distribution was observed between camps participants with both surveys and sub-groups (p<0.001 for the four groups and sub-groups).

**Table 4:** EquityTool 2010 wealth quintiles of CATCH camps participants, DHS and MIS survey individuals, at national level and Kasungu district level (weighted).

Equity tool 2010	Camps participant 2017 (n = 1,358) N (%)	DHS survey 2015/2016–Kasungu (n = 1,276) N (%)*	DHS survey 2015/2016–National (n = 24,799) N (%)*	MIS survey 2012–Kasungu (n = 145) N (%)*	MIS survey 2012–National (n = 3,404) N (%)*
Relative wealth quintiles	Ref.	P value: 0.000	P value: 0.000	P value: 0.000	P value: 0.000
Q1 poorest	125 (9.2)	42 (4.24)	549 (2.22)	60 (30.04)	771 (22.65)
Q2	219 (16.13)	73 (7.41)	981 (3.97)	51 (25.5)	679 (19.95)
Q3	322 (23.71)	342 (34.94)	7840 (31.71)	34 (16.95)	640 (18.8)
Q4	380 (27.98)	316 (32.3)	8426 (34.08)	34 (17.11)	636 (18.69)
Q5 richest	312 (22.97)	206 (21.1)	6924 (28.01)	21 (10.39)	678 (19.91)

* STATA complex sampling design F tests of independence with camps participant (p < 0.001)

#### Disability status

Using the recommended Washington Group definition of disability, 373 persons were classified as having a disability (27.5%) (Table 5). Comparing with the SINTEF national survey, the proportion of people with disabilities was significantly higher than the general population (5.6%). Looking at the differences by age group, to adjust for the different sample and population age distribution, the proportion of reported disability is consistently higher among camp participants than among the national survey population (Table 5).

**Table 5:** Comparison of sample prevalence of reported disability and SINTEF disability national survey 2017.

	Age group distribution		Prevalence of reported disability		
Age groups	Camps N(%)	SINTEF 2017 N(%)	Camps N(%)	SINTEF 2017 N(%)	X^2^ test
<20	136(10.0)	74,516(57.4)	28(20.6)	2,324(3.1)	<0.001
20–29	130(9.6)	20,939(16.1)	15(11.5)	1,046(5.0)	<0.001
30–39	190(14.0)	14,276(11.0)	32(16.8)	847(5.9)	<0.001
40–49	257(18.9)	8,456(6.5)	55(21.4)	736(8.7)	<0.001
50–59	231(17.0)	4,747(3.7)	69(29.9)	679(14.3)	<0.001
60–69	237(17.5)	3,607(2.8)	84(35.4)	633(17.5)	<0.001
70+	177(13.0)	3,306(2.5)	90(50.8)	1,047(31.7)	<0.001
**Total**	1,358(100)	129,847(100)	373(27.5)	7,132(5.6)	<0.001

Excluding the vision domain, 193 (14.2%) participants were considered having a disability, in one domain or more. The most common non-visual disabilities reported were difficulties in walking (6.6% prevalence) and difficulties in remembering/concentrating (5.8%) (S2 Table).

## Discussion

This study assessed the equity of access to eye care services of the CATCH eye health camps. Data were collected from a random sample of people presenting at the outreach camps in one of the districts in Central Malawi. We were specifically interested in patients’ socio-economic and disability status and used existing standardised international tools to collect these variables. We observed reported disability status, gender and education level, and for socio-economic status we measured both absolute poverty rates and relative wealth.

The study also helped to better understand the use of the tools for measuring patients’ socio-economic status in a routine service delivery setting. The EquityTool and the Simple Poverty Scorecard used in this study rely on asset-based wealth indexes which are considered as best practices for household surveys, where dwelling characteristics and ownership of durable assets can be directly observed. There were concerns about the reliability of self-reported data, but this study found a high level of agreement (80%) between the information given by respondents at the point of service delivery and what was observed during the follow-up visits to their homes. This suggests that self-reported data on dwelling characteristics and assets can be a reliable measure of socio-economic wealth and that the tools, such as the EquityTool and the Simple Poverty Scorecard, can be applied to collect data from participants presenting at clinics or outreach camps. Both questionnaires are simple and fast to administer (10 to 15 minutes) causing minimal disruption to health care services.

Moreover, we found that using a set of selected tools to routinely monitor equity in service delivery is feasible; and although data collection did require additional human resources, five data collectors working 24 days, the study was integrated in the ongoing programmatic activities, so allowances for drivers, district health officials, stationery and communication costs would ordinarily be covered the programme delivery costs. Moreover, data collectors’ work was neither intrusive nor disruptive to the work of the medical personnel. Indeed, CATCH staff who worked alongside the data collection team confirmed that surveying study participants had minimal effect on their usual camp routine. Collecting data from a sample of patients at more convenient time rather than all camp attendees required longer presence of data collectors at the camp but it prevented interference with the flow of patients. Collecting additional data after the initial screening was the most optimum point, as by that time the patients had received the service they came for as they were either on their way home (if they did not have any eye problems) or were waiting at the camp for further examination and treatment.

In terms of absolute wealth measurement, the Simple Poverty Scorecard results showed that camp participants’ average poverty rate was significantly higher than the poverty rates of Kasungu and national population of Malawi in 2011 [21]. The camp participants’ poverty rate was also higher than the national poverty rate measured in 2017 [24]. However, the poverty rates were similar to the levels in Kasungu. The findings suggest that the programme reached poor people in a proportion similar to the Kasungu population. The relative wealth measurement and the EquityTool results indicate that camp attendees were relatively wealthier compared to the 2012 national population and Kasungu district [22]. However, compared to the DHS 2017, the camp attendees appeared to be relatively poorer than the Kasungu and national populations, which is typical for trachoma endemic areas as trachoma is known to be a disease of poverty affecting the poorest and most marginalised communities [39].

We found the use of both tools necessary and complementary, as the Simple Poverty Scorecard shows how poor your target population is in absolute terms, while the EquityTool assesses programme populations in comparison with the national or the urban population of a country. However, it is important to note that the comparison of our camp attendees with the population of Kasungu rather than the national population produced more relevant results to measure equitable access of a local health programme. The finding suggests the importance of using regional and district level subsets of national survey data as benchmarks for comparison.

It is also important to note that the latest versions of the tools available to us at the time of the study (in 2017) were based on relatively old surveys (MIS 2012 and IHS 2010/2011); the tools have since been updated based on more recent surveys (DHS 2015–16 and IHS 2017). This is likely to explain the fact that our participants were generally wealthier when compared to the national and Kasungu district populations in 2010–11 or 2012 (as compared to more recent surveys). The results confirm an earlier observation made by Wilunda et al. that tools based on the ownership of assets tend to lose their reliability over time [32]. It is particularly true for the ownership of assets such as radios, televisions, or mobile phones, which can rapidly change over a short period of time. Therefore, to determine the socio-economic status more accurately it is essential to use the most recent household survey benchmarks and tools [40]. This finding is important to consider when integrating equity measurement in a development project cycle. For example, in the contexts where only a relatively old (five years or more) tool for measuring wealth is available, it may be better to wait for the release of an updated tool, as the conclusions based on the data from the old tool are likely to be inaccurate and misleading.

The estimated prevalence of functional limitations among programme participants attending the outreach camps was 27.5%, including all domains, and 14.2%, excluding the sight domain when using the Washington Group definition of disability. Disability data collected at the facility level is difficult to interpret, as people coming to the facilities are not necessarily representative, indeed, people coming to eye care services, are usually older and have visual impairments. However, we could compare our data with a recent survey from SINTEF of living conditions of people with disabilities in Malawi published in 2018by age group [36]. The survey used the same WGSS tool and collected disability data on a large sample of over 120,000 people. The statistical test comparing the prevalence of disability per age group showed that throughout all sub-groups there were significant prevalence differences, suggesting that camp participants were more likely to report a disability compare than the national representative survey, and hence that the CATCH programme successfully reached out persons with disability (Table 5).

Another aspect of equity that may require further attention is gender equity. Outreach camps in the CATCH programme targeted primarily people with cataract and trachomatis trichiasis. For both conditions, women are at higher risk than men. Yet, 54% of our study participants were male. Gender inequities in accessing eye health services have been well documented [9, 41–45]. In most low-income settings in Sub-Saharan Africa, women have lower coverage with cataract services and are more likely to be blind and severely visually impaired than men. The reasons for this are multiple and complex, ranging from cultural norms that value males over females to women’s inability to pay or travel outside their community [44–46]. Our study suggests that women are not only less likely to accept and access treatment, but that they are less likely to attend the first point of contact with a healthcare provider, which is outreach camps. Further studies are needed to explore the drivers of women’s health seeking behaviour and to better understand whether women do not have access to information about outreach camps or they do not come due to household, childcare, or other duties.

There are several challenges and limitations that need to be considered when interpreting these results or when planning similar studies in other locations. First, our conclusions are limited to one location, Kasungu, and cannot be generalised to other settings doing similar programmes in Malawi or elsewhere. Studies in other parts of Malawi and other countries would be beneficial to review equity in accessing eye health services in a more systematic way and whether there are any cross-country generalisations. Second, in this study we did not have access to clinical data. The data collected here was only on the attendance of outreach camps. We do not know whether there were differences in the severity of visual impairment presented at the camp or the uptake of referrals by gender, socio-economic status, or disability.

Finally, although the residence of programme participants (village and traditional authority area) was recorded in this study, it could not be classified into urban or rural as per the DHS. We did not map patients’ residence in relation to the location of the camps and did not assess whether there were any specific locations that could not be reached due to distance. Future outreach camps need to collect more accurate data on attendees’ residence and distance travelled, possibly using GIS maps. This will help to better understand the intersectionality of wealth, rural residence, gender, and disability.

## Conclusions

Despite some limitations the study shows that the outreach camps managed to reach a poor section of the district population as well persons with disabilities. However, women may find it more difficult to access the camps and need to be given a particular attention in the community mobilisation campaigns. The study also shows that self-reported data on household characteristics and assets are reliable and can be collected in clinical settings without a need for onsite observations. However, to produce accurate results on the household wealth the most recent surveys and regional datasets should be used for the purpose of benchmarking. The study provides valuable information on the tools that could be used in programme settings to assess the equity of access to eye care services. It shows that it is feasible to use the Simple Poverty Scorecard, the EquityTool and the Washington Group Short Set of Questions on Disability in an outreach camp without major disruptions to the services provided. The study further emphasises the need for monitoring equity in eye care programmes with additional data on patient residence, clinical diagnoses, and the uptake of referrals.

## Supporting information

S1 Appendix Questionnaire. (PDF)

S2 Appendix Information and consent forms. (PDF)

S1 Dataset Camp level dataset. (XLSX)

S2 Dataset Household level dataset. (XLSX)

S1 Fig Kasungu DHS participants data comparing EquityTool 2010 and EquityTool 2015 proxies. (PDF)

S1 Table Kappa individual question variables, comparison between camp survey and household survey. (PDF)

S2 Table Distribution of reported disability by domain. (PDF)

## Abbreviations

CATCH: Co-ordinated Approach To Community Health
DFID: Department for International Development
DHS: Demographic and Health Surveys
IHS: Integrated Household Survey
iNGO: international Non-Governmental Organisation
MIS: Malaria Indicator Survey
NHSRC: National Health Sciences Research Committee
PPP: Purchasing Power Parity
QEDJT: Queen Elizabeth Diamond Jubilee Trust
RAAB: Rapid Assessments of Avoidable Blindness
SDGs: Sustainable Development Goals
SSA: Sub-Saharan Africa
TT: Trachoma Trachomatous
WGSS: Washington Group Short Set
