# Supplementary material for: Measuring equity of access to eye health outreach camps in rural Malawi
Source: PLoS One. 2022 May 20;17(5):e0268116. doi: 10.1371/journal.pone.0268116 (PMC9122225; doi:10.1371/journal.pone.0268116)
Supplement: S1 Appendix — (PDF) [file pone.0268116.s001.pdf]

Questionnaire (English)

Demographics

Q1 How old are you?

Q2 Gender (Select only one.)

A. Female
B. Male
C. Other

Q3.1 What is the name of your village? [ ]

Q3.2 What is your traditional authority area? [ ]

Q3.3 Are you the head of your household?

A. Yes
B. No, If No. What is your relationship to the head of the household?

Equity Tool and Poverty scorecard

Now I am going to ask you questions about your household

Q4 Does your household have … a mobile telephone?

A. Yes
B. No

Q5 …a television?

A. Yes
B. No

Q6 … electricity?

A. Yes
B. No

Q7 …a radio?

A. Yes
B. No

Q8 … a telephone (landline)?

A. Yes
B. No

Q9 … a refrigerator?

A. Yes
B. No

Q10 Does any member of your household own… a bicycle?

A. Yes
B. No

Q11 … a motorcycle or motor scooter?

A. Yes
B. No

Q12 … a car or truck?

A. Yes
B. No

Q13 Does any member of this household have a bank account?

A. Yes
B. No

Q14 What is the main source of drinking water for members of your household?

A. Piped into dwelling
B. Piped into yard/plot
C. Public tap /standpipe
D. Tube well or borehole
E. Unprotected dug well
F. Unprotected Spring
G. Surface water-river, lake, dam, pond
H. Other

Q15 What kind of toilet facility do members of your household usually use?

A. Flush or pour flush toilet
B. Ventilated Pit Latrine (VIP) latrine
C. Pit latrine without slab/ open pit
D. Pit latrine with slab
E. No facility/bush/field
F. None, traditional latrine without roof shared with other households
G. Traditional latrine without roof only for household members
H. Traditional latrine without roof shared with other households
I. Traditional latrine with roof only for household members
J. Other

Q16 Do you share this toilet facility with other households?

A. Yes
B. No

Q17 What type of fuel does your household mainly use for cooking?

A. Electricity
B. Wood
C. Charcoal
D. Other

Q18 What is the main material of the floor in your household?

A. Cement
B. Ceramic tile
C. Earth
D. Sand
E. Dung
F. Smoothed mud
G. Wood
H. Tile
I. Other

Q19 What is the main material of the exterior walls in your household?

A. Stone walls with lime/cement
B. Unburnt bricks
C. Cane/palm/trunks/dirt
D. Bamboo/tree trunks with mud
E. Cement
F. Burnt bricks
G. Mud (yomata) or grass

H. Compacted earth (yamdindo)
I. Burnt bricks
J. Concrete
K. Wood
L. Iron sheets
M. Other

Q20 What is the main material of the roof in your household?

A. Thatch/palm leaf
B. Palm /bamboo
C. Iron sheet
D. Grass
E. Plastic sheeting
F. Clay tiles
G. Concrete
H. Other

Q21 How many members does the household have?

A. Seven or more
B. Six
C. Five
D. Four
E. One, two, or three

Q22 Is the (oldest) female head/spouse able to read and write in Chichewa or English?

A. No
B. Yes, only Chichewa
C. Yes, English (regardless of Chichewa)
D. No, female head/spouse

Q23 What is the household's main source of lighting fuel?

A. Collected firewood, purchased firewood, grass, or gas
B. Paraffin or other
C. Battery/dry cell (torch), candles, or electricity

Q24 Does any member of this household sleep under a bet net to protect against mosquitoes at some time during the year?

A. Yes
B. No

Q25 Does the household own any tables?

A. Yes
B. No

Q26 Does the household own any beds?

A. Yes
B. No

Washington Group questions

Q27 Do you have difficulty seeing, even if wearing glasses?

A. No - None
B. Yes – Some difficulty
C. Yes – A lot of difficulty
D. Yes – Cannot do at all

Q28 Do you have difficulty hearing, even if wearing a hearing aid?

A. No - None
B. Yes – Some difficulty
C. Yes – A lot of difficulty
D. Yes – Cannot do at all

Q29 Do you have difficulty walking or climbing steps?

A. No - None
B. Yes – Some difficulty
C. Yes – A lot of difficulty
D. Yes – Cannot do at all

Q29 Do you have difficulty remembering or concentrating?

A. No - None
B. Yes – Some difficulty
C. Yes – A lot of difficulty
D. Yes – Cannot do at all

Q30 Do you have difficulty (with self-care) such as washing all over or dressing?

A. No - None
B. Yes – Some difficulty
C. Yes – A lot of difficulty
D. Yes – Cannot do at all

Q31 Using your usual (customary) language, do you have difficulty communicating, for example understanding or being understood?

A. No - None
B. Yes – Some difficulty
C. Yes – A lot of difficulty
D. Yes – Cannot do at all

What is the service was offered to the respondent [Question to data collector]

Questionnaire (Chichewa)

Q1 Muli ndi zaka zingati?

Q2 Chibadidwe cha oyankha

A. Mwamuna
B. Mkazi
C. Ena

Q3 Dzina la mudzi wanu ndi chani?

Ndinu mwini khomo?

A. Inde
B. Ayi. Ngati ayi, pali ubale wanji ndi mwini khomo?

Q4 Mnyumba mwanu muli ndi … foni ya m’manja?*

A. Inde
B. Ayi

Q5 …nanga wailesi ya kanema?*

A. Inde
B. Ayi

Q6 … nanga magetsi?*

A. Inde
B. Ayi

Q7 …nanga wailesi?*

A. Inde
B. Ayi

Q8 … nanga lanya (thenifolo ya mnyumba)?*

A. Inde
B. Ayi

Q9 … nanga filiji?*

A. Inde
B. Ayi

Q10 Alipo wachibale mnyumba mwanu yemwe ali ndi… njinga ya kapalasa?*

A. Inde
B. Ayi

Q11 … nanga njinga ya moto?

A. Inde
B. Ayi

Q12 … nanga galimoto ya mtundu wina ulionse?*

A. Inde
B. Ayi

Q13 Alipo wachibale mnyumba mwanu yemwe ali ndi akaunti ya ku banki?*

A. Inde
B. Ayi

Q14 Kodi madzi omwe m'mamwa amachokera kuti?*

A. madzi ampopi ofika mnyumba
B. madzi ampopi ofika m'nkhonde
C. mpopi wa gulu
D. mjigo
E. Chisime chosatetezedwa
F. Kasupe osatetezedwa
G. madzi am'mitsinje, m'nyanja, madamu ndi zithapwi
H. zina

Q15 Kodi achibale mnyumba mwanu amagwiritsa ntchito chimbuzi cha mtundu wanji?*

A. Chimbuzi chamadzi chogujumula
B. Chimbuzi chokumba chamakono (chokhala ndi paipi yotulusira mphweya)
C. Chimbuzi chokumba chopanda silabu
D. Chimbuzi chokumba cha silabu
E. Alibe chimbuzi/kutchire/m'minda
F. Zina

Q16 M'magwiritsa chimbuzi chanu ndi anthu ochokera m'makomo ena?*

A. Inde
B. Ayi

Q17 Kodi m'maphikira chiani?*

A. Magetsi
B. Nkhuni
C. Makala
D. Zina

Q18 Mnamangira chiani pasi pa nyumba yanu?*

A. Simeti
B. Matailosi
C. Dothi, mchenga, ndowe
D. Zina

Q19 Kodi Chikupa cha nyumba yanu ndi chomangidwa ndi chiani?*

A. Chikupa cha miyala ndi laimu/simeti
B. Njerwa zosawotcha
C. timitengo/dothi
D. Bango/mapolo ndi dothi
E. Simeti
F. Njerwa zowotcha
G. Zina

Q20 Denga la nyumba yanu mnamangira chiani?*

A. Masamba/masamba amikoma
B. Mikoma/ bango /udzu

C. Malata
D. Zina

Q21 Mulimo angati mnyumba mwanu?

A. Asanu ndi awiri kapena kupitirira pamenepo
B. Asanu ndi mmodzi
C. Asanu
D. Anayi
E. Mmodzi, awiri kapena atatu

Q22 kodi mayi wapanyumbapo amatha kuwerenga ndi kulemba Chichewa kapena Chingerezi?

A. Ayi
B. Inde, Chichewa chokha
C. Inde, Chingerezi (posawerengera Chichewa)
D. Palibe mayi

Q23 Mmawunikira china mnyumba mwanu?

A. Khuni zotola, khuni zogula, uzu, phepo
B. Parafini ndi zina
C. Tochi wa mabatile, kandulo, magetsi

Q24 pali wachibale yemwe amagona muneti thawi yina poziteteza ku udzudzu?

A. Inde
B. Ayi

Q25 Muli ndi tebulo mnyumba mwanu?

A. Inde
B. Ayi

Q26 Muli ndi mabedi mnyumba mwanu?

A. Inde
B. Ayi

Q27 Kodi m’mavutika kuyang’ana, ngakhale mtavala magalasi?

A. Ayi – palibe vuto
B. Inde – pali vuto pang’ono
C. Inde – pali vuto lalikulu
D. Sindingathe ndi pang’ono pomwe

Q28 Kodi m’mavutika kuva, ngakhale mtavala zothandizira kuva?

A. Ayi – palibe vuto
B. Inde – pali vuto pang’ono
C. Inde – pali vuto lalikulu
D. Sindingathe ndi pang’ono pomwe

Q29 Kodi m’mavutika kuyenda kapena kukwera masitepe?

A. Ayi – palibe vuto
B. Inde – pali vuto pang’ono

C. Inde – pali vuto lalikulu
D. Sindingathe ndi pang'ono pomwe

Q30 Kodi m'mavutika kukumbuka zithu kapena kuvesera munthu akamakuyakhulani?

A. Ayi – palibe vuto
B. Inde – pali vuto pang'ono
C. Inde – pali vuto lalikulu
D. Sindingathe ndi pang'ono pomwe

Q31 Kodi m'mavutika kuzisamalira monga kusamba ndi kuvala nokha?

A. Ayi – palibe vuto
B. Inde – pali vuto pang'ono
C. Inde – pali vuto lalikulu
D. Sindingathe ndi pang'ono pomwe

Q32 Kodi m'mavutika kuyakhulana ndi azanu mchiyakhulo chanu (monga kuvesetsa azanu kapena azanu kukuvesetsani)?

A. Ayi – palibe vuto
B. Inde – pali vuto pang'ono
C. Inde – pali vuto lalikulu
D. Sindingathe ndi pang'ono pomwe

Questionnaire (Tumbuka)

Q1 Muli na vyaka viringa?

Q2. Chilengiwa cha wakuzgola

A. Mwanalume
B. Mwanakazi
C. Wanyakhe

Q3 Zina la chikaya chinu ni vichi?

Ndimwe mwenecho nyumba?

A. Enya
B. Yayi. Pala yayi, pali ubale wuli na mwenecho nyumba?

Q4 Munyumba mwinu muli na… foni ya mumawoko?*

A. Enya
B. Yayi

Q5 …paji wayilesi ya kanema?*

A. Enya
B. Yayi

Q6 … paji magesi?*

A. Enya
B. Yayi

Q7 …paji wayilesi?*

A. Enya
B. Yayi

Q8 … paji thelefoni ya munyumba?*

A. Enya
B. Yayi

Q9 … paji fuliji?*

A. Enya
B. Yayi

Q10 Muli na wachibale munyumba yinu uyo wali na… njinga ya kapalasa?*

A. Enya
B. Yayi

Q11 … paji njinga ya moto?*

A. Enya
B. Yayi

Q12 … paji galimoto ya mutundu wuliwose?*

A. Enya
B. Yayi

Q13 Muli na wachibale munyumba yinu uyo wali na akawunti ya ku banki?*

A. Enya
B. Yayi

Q14 Kasi maji ayo mukumwa yakufumira kochi?*

A. maji yamumupopi yakufika munyumba
B. maji yamumupopi yakufika munkhonde
C. mupopi wa gulu
D. mujigo
E. Chiziwa chambula kupwerereka
F. Kawiluwilu wambula kupwerereka
G. maji ya mumironga, munyanja, madamu, vithapwi
H. Vinyakhe

Q15 Kasi mukuluta ku chimbuzi cha mutundu wuli panyumba pinu?*

A. Chimbuzi chamaji chakugujumula
B. Chimbuzi chakujima (chakuwa na paipi yakufumiskira phepo)
C. Chimbuzi chakujima chambula silabu
D. Chimbuzi chakujima cha silabu
E. Walije chimbuzi/kuthondo/muminda
F. Vinyakhe

Q16 Mukugwirisa chimbuzi chinu na wanthu wanyakhe wakufuma zinyumba zinyakhe?*

A. Enya
B. Yayi

Q17 Kasi mukuphikira vichi?*

A. Magesi
B. Nkhuni
C. Makala
D. Vinyakhe

Q18 Kasi mulikuzengera vichi pasi panyumba yinu?*

A. Semeti
B. Matayilosi
C. Dongo, muchenga, ndope/ulongwe
D. Zina

Q19 Kasi chiwumba cha nyumba yinu mulikuzengera vichi?*

A. Chiwumba cha malibwe na layimu/semeti
B. Njerwa zambula kotcha
C. tumakuni/dongo
D. Matete/mapolo na dongo
E. Semeti
F. Njerwa zakuwotcha
G. Vinyakhe

Q20 Nyumba yinu muli kufolera na vichi?*

A. Mahamba/mahamba ya mikoma
B. Mikoma/ matete /utheka

C. Malata
D. Vinyakhe

Q21 Mulimo walinga munyumba yino?

A. Wakhonde na wawiri
B. Wakhonde na umoza
C. Wakhonde
D. Wanayi
E. Umoza, wawiri paji watatu

Q22 Kasi mama wa panyumba apa wakumanya kuwerenga na kuremba Chichewa paji Chizungu?

A. Yayi
B. Enya, Chichewa pera
C. Enya, Chizungu (kwambula kuyowoyapo va Chichewa)
D. Mulije mama

Q23 Mukuwunikira vichi mynyumba yinu?

A. Nkhuni sakusola, nkhuni zakugula, utheka, phepo
B. Parafini na vinyakhe
C. Tochi wa batire, kandulo, magetsi

Q24 Muli wachibale munyumba yinu uyo wakugona muneti nyengo zinyakhe pakunjivikirira ku nyimbo?

A. Enya
B. Yayi

Q25 Muli na thebulo munyumba yinu?

A. Enya
B. Yayi

Q26 Muli na bedi munyumba yinu?

A. Enya
B. Yayi

Q27 Kasi mukusuzgika kuwona, nangawuli mungavwala magalasi?

A. Yayi – khusuzgika yayi
B. Enya – khusuzgika pachoko
C. Enya – khusuzgika chomene
D. Khutondekerathu napachokowuwo

Q28 Kasi mukusuzgika kupulika, nangawuli mungavwala vyakuvwira kupulika?

A. Yayi – khusuzgika yayi
B. Enya – khusuzgika pachoko
C. Enya – khusuzgika chomene
D. Khutondekerathu napachokowuwo

Q29 Kasi mukusuzgika kwenda nakukwera masitepu?

A. Yayi – khusuzgika yayi

B. Enya – khusuzgika pachoko
C. Enya – khusuzgika chomene
D. Khutondekerathu napachokowuwo

Q30 Kasi mukusuzgika kukumbukira vithu na kupulikizganga pala wakukuyowoyeskani?

A. Yayi – khusuzgika yayi
B. Enya – khusuzgika pachoko
C. Enya – khusuzgika chomene
D. Khutondekerathu napachokowuwo

Q31 Kasi mukusuzgika kujipwererera (nga kungeza na ku vwala)?

A. Yayi – khusuzgika yayi
B. Enya – khusuzgika pachoko
C. Enya – khusuzgika chomene
D. Khutondekerathu napachokowuwo

Q32 Kasi mukusuzgika kuyowoyeskananga na wanyinu muchiyowoyero chinu (nga kupulikiska ivo wanyinu wakuyowoya paji wanyinu kuti wamupulikiskeni ivo mukuyowoya)?

A. Yayi – khusuzgika yayi
B. Enya – khusuzgika pachoko
C. Enya – khusuzgika chomene
D. Khutondekerathu napachokowuwo
