# Supplementary material for: Measuring equity of access to eye health outreach camps in rural Malawi
Source: PLoS One. 2022 May 20;17(5):e0268116. doi: 10.1371/journal.pone.0268116 (PMC9122225; doi:10.1371/journal.pone.0268116)
Supplement: S2 Appendix — (PDF) [file pone.0268116.s002.pdf]

HEAD OF HOUSEHOLD INFORMATION SHEET AND CONSENT

Hello. My name is ______________________________.I work with Sightsavers. Sightsavers works with partners in developing countries to treat and prevent avoidable blindness, and promote equality for people with visual impairments and other disabilities. We are conducting a study that will help us to understand how best Sightsavers is serving the community particularly whether our services are accessible by those at most need. The information will be used to improve our programs in Malawi.

Your ______________________________attended the eye health camp held in [Karoga/Kasungu] and participated voluntarily in the study. Participation in the study entails completion of a short questionnaire and a household visit. During this visit I will repeat some sections of the questionnaire that inquire about items in your household. This information is confidential and will help us understand the community we serve and also help us ensure that we provide services to all those who need it the most. As the head of this household, we require your consent to be able to proceed with this household visit. Your consent for this visit is voluntary and you can withdraw from the home visit after having agreed. . Do you have any questions? [Data collector answers all questions]. If I have answered all your questions about this household visit do you agree to also allow me to proceed with the questionnaire? Either you or [insert name of patient] can respond to the questions. Yes ____ No ____

[If respondent doesn't consent to household visit, data collector will thank him/her for his/her time and leave the household]

In case you need more information about the survey, you may contact the person listed on this card [.

[GIVE CARD WITH CONTACT INFORMATION.]

In case of you need more information about the survey, please contact:
Effie Kaminyoghe
Sightsavers International Malawi Country Office - +26501750453, Kang'ombe House City Centre, 3rd Floor, Robert Mugabe Crescent. P/Bag A197

PARTICIPANT INFORMATION SHEET AND CONSENT

Hello. My name is ______________________________.I work with Sightsavers. Sightsavers works with partners in developing countries to treat and prevent avoidable blindness, and promote equality for people with visual impairments and other disabilities. We are conducting a study that will help us to understand how best Sightsavers is serving the community particularly whether our services are accessible by those at most need. The information will be used to improve our programs in Malawi.

We would like to invite you to participate in the study. If you choose to participate, I will ask you some questions about your household and other topics. The questions usually take about 10 to 15 minutes. You don't have to be in the survey, but we hope you will agree to answer the questions since your views are important. If I ask you any question you don't want to answer, just let me know and I will go on to the next question or you can stop the interview at any time. If you choose to not participate or to withdraw your participation, your access to services in the eye camp will not be affected. All the information we collect will be kept confidential. Do you have any questions? [Data collector addresses all questions]. If I have answered all your questions do you agree to participate in the study? Yes ____ No ____

You may also be chosen for a household visit. This means that we will travel back to your home together. During this visit I will repeat some sections of the questionnaire that inquire about items in your household. To be able to do this I would like you to provide additional consent. Your consent for this visit is voluntary and you can withdraw from the home visit after having agreed. If you choose to not participate in the household visit or to withdraw your participation, your access to services in the eye camp will not be affected Providing consent indicates that you understand and will provide us with your full address, and are willing to have a home visit conducted. Do you have any questions? [Data collector answers all questions]. If I have answered all your questions about the household visit do you agree to also allow me or my colleague to come with you to your home ? Yes ____ No ____

[If respondent doesn't consent to household visit, data collector will assure him/her that he/she will not be considered for the visit].

In case you need more information about the survey, you may contact the person listed on this card.

[GIVE CARD WITH CONTACT INFORMATION

In case of you need more information about the survey, please contact:
Effie Kaminyoghe
Sightsavers International Malawi Country Office - +26501750453, Kang'ombe House City Centre, 3rd Floor, Robert Mugabe Crescent. P/Bag A197

Participant information sheet and consent form (Chichewa)

ZOMUFOTOKOZERA WOTENGAPOMBALI NDI CHIROLEZO CHAKE

Moni. Dzina langa ndi________________. Ndimagwira ntchito ku Sightsavers omwe amagwira ntchito mmayiko omwe akutukuka kumene mogwirizana ndi mabungwe ena pochiza ndi popewa matenda opangisa kusawona ndi polimbikisa ufulu wa anthu avuto la maso ndi kulumala kwina. Tikupanga kafukufuku wotithandiza kuziwa momwe Sightsavers ikugwirira ntchito makamaka ngati ntchito zathu zikufikira anthu ovutika. Zosatira zake zithandiza kukoza momwe timagwirira ntchito m'Malawi.

Tikukupephani inu kuti mutatengapo mbali mkafukufuku uyu. Mukavomera, ndikufusani mafuso okhuzana ndi pakhomo panu ndi nkhani zina. Mafuso amatenga pakati pa phindi khumi kufikira khumi ndi zisanu. Sindinu wokakamizidwa kuyakha mafusowa, koma tiri ndi chikulupiriro kuti muyakha chifukwa mayakho anu ndiwofunika. Ngati pali fuso loti simukufuna kuyakha, ndiwuzeni ndipo ndifusa lina ndiposo mukhoza kuyimisa mafuso thawi yina yiriyonse. Mukatero sikuti mutaya mwayi wolandira chithandizo mmisasa yathu. Mayakho omwe tipeze akhala achisisi. Muli ndi fuso lina lirirose? (Wopanga kafukufuku ayakha mafuso onse). Ngati ndayakha bwino mafuso anu onse, ndinu pano okozeka kutengapo mbali mkafukufukuyu? Inde _____ Ayi _______.

Mukhoza kusakhidwaso kuti tikuyendereni pakhomo panu. Zikatero ndekuti tipita limodzi kwanu. Pokuyenderani, ndibwereza mafuso okhuzana ndi zomwe muli nazo pakhomo panu. Ndipofunika chirolezo chanu chapadera kuti izi zitheke. Muli ndi ufulu kuvomereza kapena kukana ndiposo mukhoza kuyimisa kukuyenderani ngakhale mutativomereza kale. Ngati mwakana kutengapo mbali mkafukufukuyu, sikuti mutaya mwayi wolandira chithandizo mmisasa yathu koma tifuna adilesi yanu ndi chirolezo choti tikhoza kukuyendera kwanu. Muli ndi fuso lina lirorose? (Wopanga kafukufuku ayakha mafuso onse). Ngati ndayakha bwino mafuso anu onse, mukuvomereza kuti ine kapena nzanga akuyendereni kwanu? Inde____ Ayi_____ (Ngati woyakha sanavomereze kuti amuyendere kwawo, wopanga kafukufuku amuuze kuti samuyendera).

Ngati muli ndi mafuso ena okhuzana ndi kafukufukuyu, mukhoza kukumana ndi munthu yemwe dzina lake liri pa kapepalapa.

(Perekani kapepalako)

Ngati muli ndi mafuso ena okhuzana ndi kafukufukuyu, mukhoza kukumana ndi:

Effie Kaminyoghe
Sightsavers International Malawi Country Office - +26501750453, Kang'ombe House City Centre, 3rd Floor, Robert Muqabe Crescent. P/Baq A 197

Head of household information sheet and consent form _ For household Visits (Chichewa)

ZOMUFOTOKOZERA MWINI KHOMO NDI CHIROLEZO CHAKE

Moni. Dzina langa ndi________________. Ndimagwira ntchito ku Sightsavers omwe amagwira ntchito mmayiko omwe akutukuka kumene mogwirizana ndi mabungwe ena pochiza ndi popewa matenda oyambisa kusawona ndi polimbikisa ufulu wa anthu avuto la maso ndi kulumala kwina. Tikupanga kafukufuku wotithandiza kuziwa momwe Sightsavers ikugwirira ntchito makamaka ngati ntchito zathu zikufikira anthu ovutika. Zosatira zake zithandiza kukoza momwe timagwirira ntchito m'Malawi.

M'bale wanu________________ anabwera ku msasa wathu wowona matenda amaso omwe unali ku (Karonga/Kasungu) ndipo anatengapo mbali mkafukufuku poyakha mafuso ndi kuvomereza kumuyendera pakhomo pake. Pokuyenderani, ndibwereza mafuso okhuzana ndi zomwe muli nazo pakhomo panu. Mayakho omwe tipeze akhala achisisi koma atithandiza kuziwa bwino anthu ammadela momwe timagwiramo ntchito ndikufikira bwino anthu omwe amafuna chithandizo chathu. Ngati inu mwini khomo, tifuna chirolero chanu kuti izi zitheke. Muli ndi ufulu kuvomereza kapena kukana ndiposo mukhoza kuyimisa kukuyenderani ngakhale mutativomereza kale. Muli ndi fuso lina lirorose? (Wopanga kafukufuku ayakha mafuso onse). Ngati ndayakha bwino mafuso anu onse okhuzana ndi kukuyenderani, mukuvomereza pano kuti ndikhoza kuyamba kufusa mafuso? Mukhoza kuyakha mafuso inuyo kapena (zina la wodwala). Inde____ Ayi_____

(Ngati woyakha sanavomereze kuti amuyendere kwawo, wopanga kafukufuku athokoza chifukwa chathawi yawo ndipo asazike).

Ngati muli ndi mafuso ena okhuzana ndi kafukufukuyu, mukhoza kukumana ndi munthu yemwe dzina lake liri pa kapepalapa.

(Perekani kapepalako)

Ngati muli ndi mafuso ena okhuzana ndi kafukufukuyu, mukhoza kukumana ndi:
Effie Kaminyoghe
Sightsavers International Malawi Country Office - +26501750453, Kang'ombe
House City Centre, 3rd Floor, Robert Muqabe Crescent. P/Baq A 197

Participant information sheet and consent form (Tumbuka)

VAKUMULONGOSOLERA WAKUFUMBIKA NA KUZOMERA KWAKE

Monile. Zina lane ni _____________. Khugwira ntchito ku Sightsavers awo wakugwira ntchito muvyalo ivo vikutukuka sono pamoza na mabungwe yanyakhe pakuchizga na kupewa chibulumutira na kulimbikiska wanangwa wa wanthu wa chibulumutira na vilima vinyakhe. Tikupanga kafufuku wakutiwovwira kumanya umo Sightsavers yikugwirira ntchito chomenechomene pala ntchito zithu zikufikira wanthu wakusuzgika. Ivotisangenge vitovwirenge kugwira makola ntchito mu Malawi.

Tikukhumba imwe kuti mupange nawo kafukufuku uyu.. Palamwazomera, nikufumbeninge mafumbo ya panyumba pinu na makani yanyakhe. Mafumbo yakutola pakati pa maminiti teni na fifitini. Ndimwe wakuchichizgika yayi kuzgola mafumbo, kweni tiri na chigomezgo kuti muzgolenge chifukwa mazgolo yinu ngakukhumbikwa. Pala pali fumbo ilo mukukhumba yayi kuzgola, niphalirani kuti nirileke nakufumba linyakhe kweniso mulinamazaza kulekeska mafumbi nyengo yiriyonse. Pala mwakana nikokuti mulekenge yayi kovwirika mumisasa yithu. Mumanyeso kuti mazgolo yinu yawenge yachisisi. Muli na fumbo lirironse? (Wakupanga kafukufuku wakuzgola mafumbo yonse). Pala nazgola makola mafumbo yinu yonse, kasi ndimwe wakunozgeka kupanga nawo kafukufuku uyu? Enya_______ Yayi_____

Mungasoleka kuti timwendereni panyumba pinu. Palaviwenge thena niko kuti tilutirenge lumoza kukwinu. Pala namwenderani, niwerezgenge mafumbo ya ivo muli navo panyumba pinu. Pakhumbikwenge chizomerezgo chinu kuti nimwendereni. Muli na ufulu kuzomera panji kukana kweniso mulinamazaza kulekeska kumwenderani. Pala mwakana kupanga nawo kafukufuku uyu, nikokuti mulekenge yayi kovwirika mumisasa yithu, kweni tikhumbenge adilesi yinu na chizomerezgo chakuti ningakuyenderani ku kwinu. Muli na fumbo lirironse? (Wakupanga kafukufuku wakuzgola mafumbo yonse). Pala nazgola makola mafumbo yinu yonse, kasi ndimwe wakunozgeka kuti ine paji munyane wamwendereni kwinu? Enya_______ Yayi_____ (Pala wakuzgola wakana kwendereka, wakupanga kafukufuku wawaphalire kuti wawayenderenge yayi).

Pala muli na mafumbo yanyakhe pa kafukufuku uyu, mungamanya kukumana na munthu uyu zina lake liri pa pepara ili.

(Perekani kapepara)

Pala muli na mafumbo yanyakhe pa kafukufuku uyu, mungamanya kukumana na:

Effie Kaminyoghe

Sightsavers International Malawi Country Office - +26501750453, Kang'ombe

House City Centre, 3rd Floor, Robert Muqabe Crescent. P/Baq A 197

Head of household information sheet and consent form _ For household Visits (Tumbuka)

VAKUMULONGOSOLERA MWENECHO NYUMBA NA KUZOMERA KWAKE

Monile. Zina lane ni _____________. Khugwira ntchito ku Sightsavers awo wakugwira ntchito muvyalo ivo vikutukuka sono pamoza na mabungwe yanyakhe pakuchizga na kupewa chibulumutira na kulimbikiska wanangwa wa wanthu wa chibulumutira na vilima vinyakhe. Tikupanga kafufuku wakutiwovwira kumanya umo Sightsavers yikugwirira ntchito chomenechomene pala ntchito zithu zikufikira wanthu wakusuzgika. Ivotisangenge vitovwirenge kugwira makola ntchito mu Malawi.

Mubale winu______________ wakiza ku musasa withu wakuwona matenda ya maso uwo ukawa ku (Karonga/Kasungu) kweniso wakapanga nawo kafukufuku pakuzgola mafumbo na kuzomera kuti timwendere kwawo. Pala namwenderani, niwerezgenge mafumbo ya ivo muli navo panyumba pinu. Mumanyeso kuti mazgolo yinu yawenge yachisisi kweni yatiwovwirenge kumanya wanthu muvikaya ivo tikugwirako ntchito na kumanya kufikira makola wanthu awo wakukhumba nadi wovwiri withu. Imwe nga mwenecho nyumba, tikukhumba chizomerezgo chinu kuti timwendereni. Muli na ufulu kuzomera panji kukana kweniso mulinamazaza kulekeska kumwenderani. Muli na fumbo liríronse? (Wakupanga kafukufuku wakuzgola mafumbo yonse). Pala nazgola makola mafumbo yinu yonse yakukhwaskana na kukwenderani, kasi ndimwe wakunozgeka kuti ningayamba kufumba mafumbo? Mungazgola ndimwe paji (zina la mulwali) Enya_______ Yayi_____

(Pala wakuzgola wakana kwendereka, wakupanga kafukufuku wawonge chifukwa cha kuwapo kwawo na kuwalayira).

Pala muli na mafumbo yanyakhe pa kafukufuku uyu, mungamanya kukumana na munthu uyu zina lake liri pa pepara ili.

(Perekani kapepara)

Pala muli na mafumbo yanyakhe pa kafukufuku uyu, mungamanya kukumana na:

Effie Kaminyoghe

Sightsavers International Malawi Country Office - +26501750453, Kang'ombe

House City Centre, 3rd Floor, Robert Muqabe Crescent. P/Baq A 197
