# Supplementary material for: Measuring equity of access to eye health outreach camps in rural Malawi
Source: PLoS One. 2022 May 20;17(5):e0268116. doi: 10.1371/journal.pone.0268116 (PMC9122225; doi:10.1371/journal.pone.0268116)
Supplement: S1 Fig — (PDF) [file pone.0268116.s005.pdf]

S5 Figure: Kasungu DHS participants data comparing EquityTool 2010 and EquityTool 2015 proxies

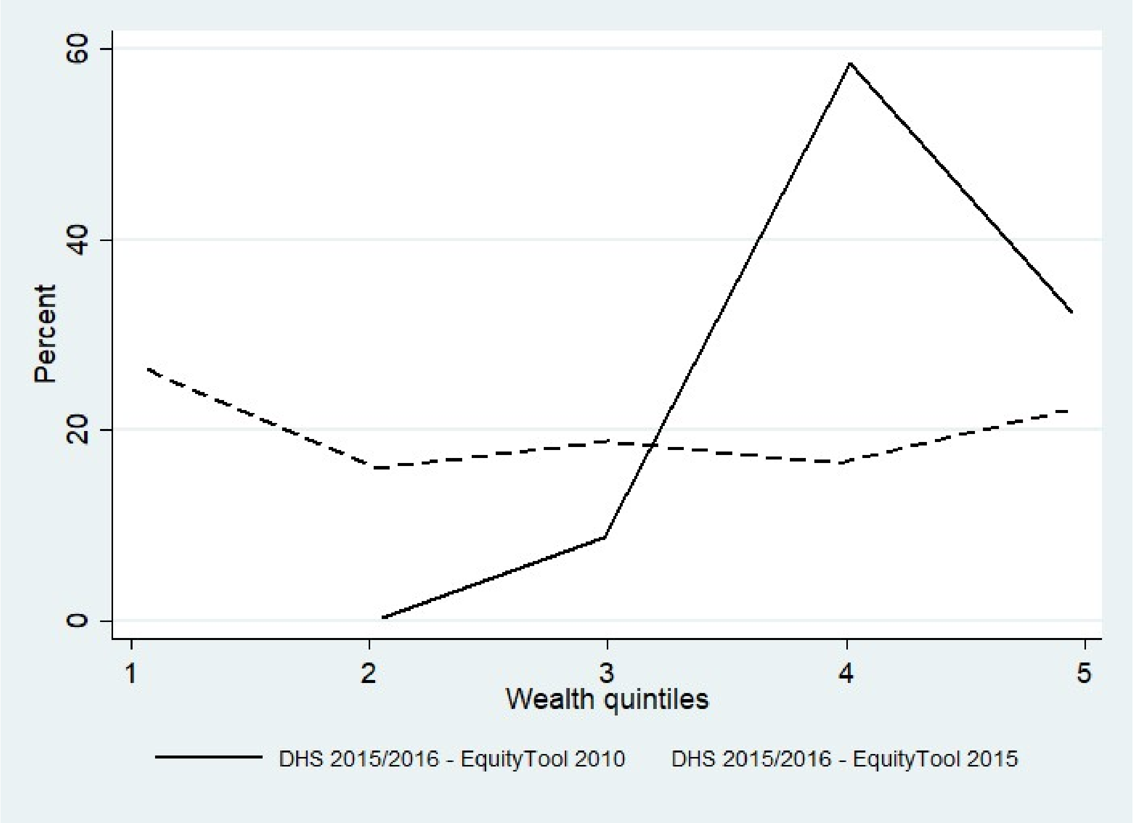

S6 Figure illustrates the differences in wealth quintiles allocation depending on proxies used. The DHS dataset (survey conducted in 2015/2016), using EquityTool proxy variables of 2010 shows that Kasungu population in 2015/2016 are relatively wealthier than in 2010, with no one belonging to the poorest quintile and almost all households belonging to the three richest quintiles. The EquityTool proxy variables of 2015 shows that Kasungu households, in terms of relative wealth, are quite representative of the national population of Malawi, as using the national cut-off points shows a balanced distribution (between 16% and 26% per quintile).
