# Supplementary material for: Measuring equity of access to eye health outreach camps in rural Malawi
Source: PLoS One. 2022 May 20;17(5):e0268116. doi: 10.1371/journal.pone.0268116 (PMC9122225; doi:10.1371/journal.pone.0268116)
Supplement: S1 Table — (PDF) [file pone.0268116.s006.pdf]

S6 Table: Kappa individual question variables, comparison between camp survey and household survey

Question	Agreement	Expected agreement	Kappa
**Q1**	94.23%	54.49	0.8732
**Q2**	94.23%	67.01	0.8251
**Q3**	98.08%	85.26	0.8696
**Q4**	92.95%	50.00	0.8590
**Q5**	99.36%	99.36	0.0000
**Q6**	97.44%	92.60	0.6537
**Q7**	87.82%	51.73	0.7477
**Q8**	98.08%	85.26	0.8696
**Q9**	96.79%	90.83	0.6505
**Q10**	94.87%	53.62	0.8894
**Q11**	91.67%	60.81	0.7874
**Q12**	89.74%	55.97	0.7671
**Q13**	86.54%	71.79	0.5227
**Q14**	96.79%	86.25	0.7668
**Q15**	94.23%	53.21	0.8767
**Q16**	92.31%	62.39	0.7955
**Q17**	96.79%	50.35	0.9355
