# Supplementary material for: Measuring equity of access to eye health outreach camps in rural Malawi
Source: PLoS One. 2022 May 20;17(5):e0268116. doi: 10.1371/journal.pone.0268116 (PMC9122225; doi:10.1371/journal.pone.0268116)
Supplement: S2 Table — (PDF) [file pone.0268116.s007.pdf]

S7 Table: Distribution of reported disability by domain (n=1,358)

Domain	Reported difficulty
See	265 (19.5%)
Hear	28 (2.1%)
Walk	90 (6.6%)
Remember	79 (5.8%)
Self-care	47 (3.5%)
Communicate	7 (0.5%)
**Individuals reporting a disability, in one domain or more**	**373 (27.5%)**
**Individuals reporting a disability, excluding vision difficulties**	**193 (14.2%)**
